# Varenicline induced auditory hallucinations in a young female with bipolar disorder: a case report

**DOI:** 10.1186/s12888-022-04348-6

**Published:** 2023-01-03

**Authors:** Vivian W. L. Tsang, Saundarai Bhanot, Lingsa Jia

**Affiliations:** 1 Department of Psychiatry, Faculty of Medicine, University of British Columbia, Detwiller Pavilion, 2255 Wesbrook Mall, BC V6T 2A1 Vancouver, Canada; 2 Department of Biochemistry and Biomedical Sciences, Faculty of Health Sciences, McMaster University, L8S 4K1 Hamilton, ON Canada

**Keywords:** Varenicline, Bipolar disorder, Hallucinations, Psychosis, Addiction, Drug induced

## Abstract

**Background:**

Creating appropriate and sustainable treatment plans for patients with concurrent disorders presents a challenge to psychiatrists and addiction medicine specialists alike. Although varenicline has been found to be the most effective medication for smoking cessation and abstinence when compared to results from placebo medications, nicotine patches and bupropion, caution is needed when starting patients on this medication. With the high prevalence of concurrent mental health and substance use disorders in vulnerably-housed populations in Canada, it becomes increasingly important to advocate for increased guidance and research into treating concurrent disorders.

**Case presentation:**

In this case, a young female patient provisionally diagnosed with bipolar I disorder was hospitalized for a manic episode in the context of substance use and medication noncompliance. She also endorsed a long history of tobacco, alcohol, cocaine, cannabis and ketamine use. Perceptual abnormalities, including auditory hallucinations, were not recorded at admission. In addition to being stabilized for bipolar diagnosis, the patient was started on nicotine replacement therapy on Day 7 of admission followed by initiation of varenicline for smoking cessation on Day 14 of admission. Soon after the varenicline treatment was started, the patient developed auditory hallucinations, paranoia and referential beliefs. However, her insight was intact, and she had minimal thought form disorganization. In this case, these symptoms were thought to be secondary to varenicline after the consideration of potential alternative contributors.

**Conclusion:**

The occurrence of side effects as a result of varenicline use in patients with diagnosed mental health conditions is rare and underlying psychiatric illness is not labeled as an absolute contraindication in the prescription of varenicline. However, it is important to advocate for increased guidance and research on the treatment of substance use disorders in patients with bipolar I disorder. Patients may also benefit from increased collaboration between psychiatric and addiction services as that may allow for earlier recognition and intervention of symptoms to minimize distress.

**Supplementary Information:**

The online version contains supplementary material available at 10.1186/s12888-022-04348-6.

## Background

Compared to the general population of Canadians, vulnerably-housed populations in Canada have a higher prevalence of concurrent mental health and substance use disorders [1]. Determining treatment plans for patients with concurrent disorders presents a challenge with respect to associated risks in such populations.

Varenicline is one of the most effective U.S. Food and Drug Administration and Health Canada approved medications used for smoking cessation. Varenicline tartrate tablets made by Pfizer Inc. [3] were approved for smoking cessation in 2007 by the Government of Canada. It works as a partial agonist to the nicotine receptor with high affinity and selectivity to α4β2 neuronal nicotinic acetylcholine receptors [4]. As per the double-blinded EAGLES trial, “varenicline-treated participants achieved higher abstinence rates than those on placebo, nicotine patch and bupropion” [5]. There has also been evidence of involvement in stimulation of dopamine receptors in the central nervous system, posing as a possible mechanism for mood-related side effects [6]. In the past, varenicline use has been linked to rare episodes of psychosis in patients diagnosed with bipolar disorder [7]. However, it is not a current absolute contraindication to prescribe varenicline in patients with underlying psychiatric illnesses [8]. It is suggested that symptoms be monitored for further deterioration as varenicline is considered to be a medication that may worsen neuropsychiatric symptoms as noted in official pharmaceutical guidelines for the medication [8]. The efficacy of varenicline for smoking cessation and rare occurrence of side effects result in prescribing practices that favor its use even amongst patients with diagnosed mental health conditions [9].

## Case presentation

This case study will discuss the hospital course of a 22-year-old shelter-housed female patient provisionally diagnosed with bipolar I disorder, hospitalized for a manic episode in the context of stimulant use and medication noncompliance to lithium. This review discusses the development of auditory hallucinations (AH) after initiation of varenicline for smoking cessation. This case is unique in that it highlights the potential risk of de-stabilization in a vulnerable youth with newly diagnosed bipolar I disorder and precarious social circumstances, in attempts to further concurrent approaches to psychiatric care.

The main objective exam used to track patient progress through the duration of her hospitalization was the mental status exam (MSE). This is standard practice for psychiatric care and qualitatively assesses factors related to a patient’s behavioral and cognitive functioning [10]. Important factors assessed for this patient include appearance and behavior, speech, affect and mood, thought form, thought content, perceptual abnormalities, insight and cognition.

### Psychiatric history

The patient reported at least five elevated mood episodes in recent years and one previous hospitalization 8 months prior to her current admission. A review of psychiatric progress notes indicates that there was disinhibition present during this previous admission in the form of oversexualized behavior and only superficial cooperation was established. It was also documented that the patient demonstrated inappropriate physical intimacy with a male patient during the admission, which was attributed to her hypomanic state. She was diagnosed with stimulant-induced psychosis thought to be secondary to cocaine use as well as bipolar disorder. By the end of a two-week period, the patient was discharged with appropriate living arrangements and a prescription for lithium that was subsequently not filled.

### Social and developmental history

The patient was raised by her biological mother until the age of 7, after which she and her sister were taken out of their mother’s custody and put into foster care. It is reported that their mother engaged in substance use, sex work, and had been in jail for severe physical attacks on one of her sexual partners. The patient also recalls her mother vaguely stating in the past that she has bipolar disorder in addition to vague mood instability.

At age 9, the patient began living with her adopted parents. While the patient states that her adoptive parents were relatively supportive and denies being in trouble at school or in other social situations, she always wanted to leave home. She left home at age 18 after graduating high school and subsequently completed two years of a business degree. Despite her initial goals of pursuing a master’s degree, she became involved with substance use in her late teenage years. With a desire to leave her home province, the patient unknowingly became part of a sex trafficking ring that brought her to the province in which this hospital admission took place. She self-described being forced to live in vulnerable housing conditions - some of which involved sexual interactions to continue residence. The patient also reported instances of forcible confinement and sexual assault.

### Substance use history

The patient’s substance use history consisted of smoking up to half a pack per day, and binge-drinking of alcohol from her teenage years approximately twice a week with an average of 12 drinks each time. She also endorsed smoking approximately one gram of cocaine per week since the age of 19, several grams of cannabis daily on a regular basis since the age of 17, as well as the use of ketamine. She described using substances for social purposes as well as a way to help her with stress.

### Treatment timeline

During the initial days of psychiatric treatment, the patient was admitted on an involuntary basis to the psychiatric stabilization unit. Perceptual abnormalities, including auditory hallucinations were not recorded during her admission consult. The patient also remained fairly paranoid about men in the community, which may be based on the reality of her past circumstances. With the patient’s date of admission being defined as Day 0, on Day 6, she stated that “my sleep [was] good” and that her mood was “much steadier”. She voiced a number of future oriented plans, stating “I want to go back to school to be a healthcare assistant” and that “I want to get my life together” and get out of the sex industry. She states that she has previously experienced hallucinations but claims she “only [heard] them when [she was] manic”. After stabilization and thorough assessment by the psychiatric team, lithium and aripiprazole treatment was started on Day 9 and Day 11 respectively for management of bipolar I disorder (Appendix 1).

She was eventually transferred to an inpatient psychiatric unit and seen by the addiction medicine service. After this assessment, she was started on nicotine replacement therapy as per her preference for smoking cessation. After further re-assessments and conversation regarding smoking cessation, the patient showed interest in starting varenicline and agreed to the treatment after discussion about the medication and potential adverse effects including neuropsychiatric side effects. Varenicline was started on Day 14. Upon assessment on Day 15, the patient had an episode of emesis during the morning and reported experiencing vivid but enjoyable dreams at night, with no other symptoms. She was provided further instructions regarding side effects and after this indicated she wished to continue treatment.

On Day 16, the patient developed auditory hallucinations, paranoia and referential beliefs. However, her insight was intact, and she had minimal thought form disorganization. Her symptoms were thought to be secondary to varenicline, and she met criteria for a substance/medication-induced psychotic disorder as per the *Diagnostic and Statistical Manual of Mental Disorders, 5th Edition* criteria. On Day 17, the patient reported hearing auditory hallucinations of a derogatory nature described as “voices of people [she] knows” (Table 1). In addition, the patient’s affect appeared to be more distressed and irritable on Day 18, compared to MSE results recorded from Day 5 to Day 15 (Table 2). During this time, the patient also expressed interest of returning to the province that she grew up in despite her family’s disagreement. Urgent reassessment occurred given the need to balance treatment of her nicotine use disorder with the risk of ongoing psychotic symptoms. After consultation between the addiction medicine and psychiatry teams, varenicline was discontinued on Day 18 and the patient was monitored for resolution of these symptoms (Table 3). In her destabilized condition, she was thought to be at high risk in community given her history of high risk sex work and sex trafficking. The original plan for discharge was therefore halted due to the vulnerability of this patient.

**Table 1:** Reports of AH during patient’s (pt) main admission

Date	Noted Auditory Hallucinations
Day 5	Patient (pt) feels safe in hospital and denies any auditory/visual hallucinations
Day 14	Denies hearing any AH and states “I only hear them when I’m manic”
Day 15	Had reportedly voiced AH to nursing staff, and she states during notes “I sometimes hear a mumble” but it did not appear to be overtly hallucinations
Day 17	AH present. Pt states voices occur with her mania. Reports voices tell her what to wear when she is deciding on an outfit to put on. Reports to hear voices while walking in the hallway. States she turns her head to look, no one is there. Reports she hears voices coming from people sitting beside her. When she looks, their mouths are not moving, and she continues to hear the voices. Pt states listening to loud music helps her cope with AH.—pt states voices are “louder”. “States she had auditory hallucinations earlier in the day of a derogatory nature-states they are voices of people she knows. Denies they are command. Reports previous medications helpful reducing them. Settled on unit. Loxapine 10 mg, Ativan 2 mg, and Seroquel 50 mg po prn with evening medications
Day 18	AH present. Pt reports AH are “quieter” than yesterday. Pt denies AH to be derogatory. AH are not command in nature. As per nursing notes, patient endorsed having an upset stomach and AH that tell her how to dress. Coping mechanism being listening to music. AH endorsed as command. Pt absconded while on a group walk. Returned on her own on the same day.
Day 19	Asked about AH today—“nothing yet today” Reports that AH “are not present this morning”- she states that she thinks they were because “of that new medication for stopping smoking that they gave me”; reports that the AH have decreased since stopping the same. No other voiced or observed perceptual disturbances present. No formal cognitive testing completed; however, the patient was attentive throughout the interview and there were no observed memory impairments present. Insight is increasingly becoming fuller as she becomes more self-aware and experiencing less manic symptoms -recognizes that her previous drug use has [led] to these current problems and that she is better without them
Day 20	Pt reports AH occurring in the middle of the day. Pt unsure of [the] same being voices or overhearing nursing staff speak. Student Nurse—Brief AH reported; however [the] same was not bothersome. From MSE—Perceptual Abnormality—Denies

Varenicline dosage was initiated on Day 14 and discontinued on Day 18 following concerns of persistent AH reported by the patient

**Table 2:** Baseline mental status examination results prior to varenicline administration

MSE Factor	Day 5	Day 6	Day 7	Day 8	Day 11	Day 12
Appearance and Behavior	Fair grooming and hygiene. No psychomotor disturbances were noted	Good rapport but somewhat superficial	Somewhat over familiar—making some facial gestures and leaning in during conversation. Fair grooming and hygiene. No psychomotor disturbances. Fair eye contact	Less overfamiliar today. Fair grooming and hygiene. No psychomotor disturbances. Fair eye contact	Good eye contact. did not appear over familiar or disinhibited today. reasonable grooming. no psychomotor agitation	Good eye contact. did not appear over familiar or disinhibited today. reasonable grooming. no psychomotor agitation
Speech	Her speech was fast and often difficult to interrupt	Soft but rapid. Not pressured. Interruptible	Speech—Soft but rapid. Not pressured. Interruptible	Soft but rapid. Not pressured. Interruptible	Rapid speech verging pressured at times	Speech less rapid, pressured today
Affect/Mood	Affect was labile, dysphoric. Mood was described as low	Mood "pretty good" and affect mildly labile. More tearful today	Affect/Mood—Mood "pretty good" and affect mildly labile. Some tearfulness	Mood "pretty good" and affect mildly labile. More irritable today	Mood 'down' affect—calm, no lability. no irritability	Mood 'better' affect—calm, mild irritability when writer was having difficulty following her mildly disorganized account of the recent months
Thought Form		Fairly organized	Initially organized though became quite tangential when discussing male acquaintance	Fairly organized	Difficult to follow events of the last few years—convoluted at times,	Convoluted at times
Thought Content	There were delusions of paranoia and referential delusions as well. Thought process was circumstantial and at times tangential. She denied any perceptual disturbances and she did not appear to be responding to any internal stimuli	No suicidal ideation (SI) or homicidal ideation (HI); no overt paranoia voiced today. (May hear [mumbling] at times but this does not sound like AH)	No SI or HI; remains paranoid about men in community (may be based in reality) Denies perceptual abnormality	No SI or HI; remains paranoid about men in community (may be based in reality) Denies perceptual abnormality	No SI or HI; remains paranoid about "pimp ring' in community (? reality based). not attending to internal stimuli during interview	No SI or HI; remains paranoid about "pimp ring' in community (? appears reality based)
Insight	Fair	Intact, superficial	Intact, superficial	Intact, superficial	Intact, superficial	Intact, superficial
Judgment	Fair	Likely poor	Likely poor	Likely poor	Slowly improving	Slowly improving
Psychiatrist l	Psychiatrist 1	Psychiatrist 2	Psychiatrist 2	Psychiatrist 2	Psychiatrist 3	Psychiatrist 3

**Table 3:** Mental status examination results during and after termination of varenicline use

MSE Factor	Day 15	Day 18	Day 19	Day 20	Day 25	Day 26
Appearance and Behavior	Good rapport but somewhat superficial	Good eye contact, did not appear overfamiliar or disinhibited today. Reasonable grooming. no psychomotor agitation	Good eye contact. Settled. Did not appear overfamiliar or disinhibited today. reasonable grooming. No psychomotor agitation	Caucasian female, blond hair and hospital gown. Looks her stated age. Wearing baggy t-shirt. Good rapport but somewhat superficial	Caucasian female, blond hair in her own clothes. Wearing an oversized sweatshirt and leggings, and hair tied back in a ponytail. appears her stated age. good eye contact. Settled. No longer appears overfamiliar or disinhibited. reasonable grooming. No psychomotor agitation	Caucasian female, blond hair in her own clothes. Dress, vest, well dressed and groomed, hair tied back in a ponytail. Appears her stated age. Good eye contact. Settled. No longer appears overfamiliar or disinhibited. reasonable grooming. No psychomotor agitation
Speech	Soft but rapid. Not pressured. [Interruptible]			Soft and less rapid. Not pressured. [Interruptible]		
Affect/Mood	Mood “pretty good” and affect mildly labile. More tearful today	Mood ‘6–7/10’ affect—distressed	Mood ‘irritable’ affect—less distressed compared to yesterday	Mood pretty good and affect much more steady today	Mood ‘stable’ affect – settled, [euthymic]	Mood ‘stable’ affect – anxious, mildly distressed
Thought Form	Fairly organized	Linear	Linear	Fairly organized	Linear	Linear
Thought Content	No SI or HI; no overt paranoia voiced today. (May hear [mumbling] at times but this does not sound like AH)	No SI or HI; AH – benign command AH, derogatory AH, referential delusions	No SI or HI; denies psychotic symptoms today (AH, TB, referential delusions)	No SI or HI; vague residual paranoia	No SI or HI; denies psychotic symptoms today (AH including mumbling, thought blocking, referential delusions). some somatic preoccupation, able to reality test	No SI or HI; AH, mumbling, thought blocking. No somatic concerns today
Insight	Intact, superficial	Able to reality test, with prompting	Able to reality test	Intact, superficial	Improved compared to last week, able to reality test	Improved compared to last week, able to reality test
Judgement	Likely poor	Limited – recent running away from group walk	Limited re: recent brief leave from group walk	Likely poor	Improving	Improving
Psychiatrist l	Psychiatrist 2	Psychiatrist 3	Psychiatrist 3	Psychiatrist 2	Psychiatrist 3	Psychiatrist 3

## Discussion and conclusions

This case highlights a challenging experience encountered by many psychiatric clinicians who navigate the concurrent treatment of psychiatric and substance use disorders. The prevalence of tobacco use disorder was found to be approximately 46.3% in those with bipolar disorder, much higher than in the general population [11]. It is necessary to be vigilant to potential adverse events from medications in particular in vulnerable demographics. This includes children and youth, patients with new or unstable psychiatric diagnoses, and the potential for destabilization due to other psychosocial reasons.

There may be alternative causes for the collection of symptoms in this patient; one being dissociative symptoms caused by trauma and stress-related challenges. However, while the patient’s previous social and developmental challenges may have played a role in her overall presentation, it is less likely that it was the primary cause for the auditory hallucinations that developed.

Stressful events during the patient’s course of admission could also have contributed to her new symptoms. For example, she absconded from a group walk on Day 14. The patient reports that there were no plans to abscond and that she walked ahead of the group in order to engage in conversation with another patient. The other patient ran towards a bridge in concern mid-conversation and she felt that she had to run after him. Though she eventually made her way back to the group and was apologetic for absconding, she states that the experience was “quite triggering” because of the proximity of the bridge to an apartment where she was reportedly trapped for a couple of months. The sudden recollection of traumatic events could have also contributed to her new auditory hallucinations, paranoia, and referential beliefs.

Furthermore, there remains the possibility that the patient may not have been adequately treated for her bipolar disorder and the auditory hallucinations was secondary to the underlying mood disorder. However, this is less likely as she had been demonstrating gradual improvement, her medication was within therapeutic range, and compliance was closely monitored, thus decreasing this likelihood. Another possibility is that she is reporting adverse effects purposefully in order to minimize her medication load. On several instances, she stated that certain medications made her nauseous. In addition, she asked many questions about particular medications on the treatment plan throughout her stay in hospital. On one occasion she also expressed that the auditory hallucinations “[a]re not real and related to that medication”, referring to varenicline.

Lastly, there are a few details of this case that are not in keeping with known literature. There have been documented cases of varenicline causing manic episodes in patients with bipolar disorder and exacerbation of schizophrenia [12, 13]. However, although these case reports have described elevated mood, pressured speech and racing thoughts, some of the symptoms experienced by our patient such as isolated auditory hallucinations and referential delusions have not been specifically reported as an adverse effect from varenicline [12, 13].

Given the timeline of the development of the patient’s new symptoms, along with unlikely alternatives, varenicline-induced psychosis remains our provisional diagnosis (Table 4). Other supportive details for varenicline-induced psychosis include the patient’s consistent demonstration of good insight of their surroundings and treatment, which is often impaired in patients with exacerbations of a primary psychiatric diagnoses [14].

**Table 4:** Timeline of important relevant events to varenicline administration and past admissions

**Previous Admission**	December 2020	Patient was transferred from another hospital where she has had brief admissions as a result of drug-induced psychosis. She was transferred to this hospital because a psychiatrist at the previous hospital felt that she was likely going through a manic phase. After assessment was considered to meet the criteria for a bipolar affective disorder. Patient was initiated on lithium carbonate which was subsequently not filled.
**Previous Discharge**	January 2021	She fully recovered from her bipolar disorder and mania had resolved. There was no evidence of hypomania or mania and no evidence of depression or suicidality.
**Main Admission**	Day 0	Patient was admitted to hospital and certified (x 2) under the mental health act. She presented to the hospital with her support worker with concerns about a foot infection and cuts from self-harm. She spoke with the ED triage and nurses about how men have been following her and very tearful when speaking about a recent episode where she says was attacked by these men. She talked about wanting a pregnancy test and STI testing because was not taking contraception. She stated that she has not been taking her bipolar medicine because it makes her feel very drowsy and does not like it. At triage, she stated that she gets very depressed, that she has overdosed on several drugs in the past and may do so again.
	Day 1	Admitting diagnoses of bipolar I disorder, mania with psychosis and polysubstance use (stimulants, opioids, unintentional opioids, nicotine). On psychiatric assessment, she presented as very disorganized and manic with flight of ideas present. She was agitated and unable to cooperate with an interview. It was clear that she would benefit from hospital stabilization based on collateral information as detailed above as well as her presentation. There is also a possible contributing factor of her increased stimulant use over the past week, specifically crystal meth.
	Day 4	Urine drug screen was positive for amphetamines, fentanyl, and benzodiazepines. Her lithium level was negligible.
	Day 5	She reports noncompliance with her lithium and crystal meth use. She feels safe in hospital and denies any auditory/visual hallucinations.
	Day 14	Varenicline dosage begun, patient reports intermittent bothersome AH, absconded from group walk to follow fellow patient out of concern.
	Day 16	Patient developed symptoms of psychosis, likely secondary to varenicline. This was characterized by auditory hallucinations, paranoia and referential beliefs with intact insight.
	Day 18	Varenicline discontinued and varenicline-induced psychosis resolved.
**Discharge**	Day 34	Patient was discharged.

It is important to advocate for increased guidance and research on the treatment of substance use disorders in patients with bipolar I disorder. This was not addressed in the recent CANMAT guidelines. Further, patients may benefit from the increased collaboration between psychiatric and addiction services as that may have allowed for even earlier recognition and intervention of the patient’s psychotic symptoms to minimize distress. In addition, nicotine use disorder in patients with mental illness is a key research area given the high comorbidity and severe medical sequelae [11].

## Supplementary Information

**Additional file 1: Appendix 1.** Medications provided to patient prior to, and after, varenicline administration.

## Data Availability

Not applicable.

## Abbreviations

AH: Auditory hallucinations
MSE: Mental status exam
Pt: Patient
